# Artificial intelligence in public health: a foundational shift or a technocratic distraction?

**DOI:** 10.3389/fpubh.2026.1789522

**Published:** 2026-03-10

**Authors:** B. Sreya

**Affiliations:** Joy University, Vaddakkankulam, Tamil Nadu, India

**Keywords:** artificial intelligence, equity, ethics, governance, population health, prevention, public health systems

## 1 Introduction

Artificial intelligence (AI) is increasingly presented as a transformative force in public health. From real time disease surveillance and outbreak forecasting to health system optimization and policy decision support, AI driven tools promise speed, scale, and analytical power beyond conventional approaches. Advances in computational capacity, coupled with the rapid expansion of digital health data including electronic health records, mobile health platforms, environmental sensors, and social media, have accelerated interest in applying machine learning and large multimodal models (LMMs) to population health challenges. Yet public health is not merely a technical or computational enterprise; it is fundamentally a normative, value driven discipline grounded in prevention, equity, and social accountability. Decisions in public health affect entire populations and often involve trade-offs that require ethical judgment, political legitimacy, and community trust. As emphasized by Wang et al. (1), the integration of AI into health systems therefore raises questions that extend beyond performance metrics: Whose data are represented? Whose values are encoded? And who remains accountable when algorithmic recommendations shape policy? This Opinion article argues that AI can represent a foundational shift for public health only if it is explicitly aligned with core public health principles. When adopted uncritically, AI risks functioning as a technocratic distraction, privileging data centric efficiency over social context and reinforcing existing structural inequities. By examining methodological tensions, normative conflicts, and governance challenges, this article seeks to contribute constructively to ongoing debates on how AI should be positioned within contemporary public health practice.

## 2 AI as a public health instrument: methodological tensions

AI applications in public health typically rely on machine learning techniques designed to detect patterns within complex, high-dimensional datasets. Compared with traditional statistical models, these approaches can capture non-linear relationships, integrate diverse data streams, and adapt dynamically as new information becomes available (2). Such capabilities are particularly attractive in settings characterized by uncertainty and rapid change, such as infectious disease outbreaks or climate-sensitive health threats. However, methodological sophistication does not automatically translate into public health relevance. A growing body of scholarship highlights the tension between predictive accuracy and interpretability. Many high-performing models operate as “black boxes,” producing outputs that are difficult to explain to policymakers, practitioners, or affected communities (16). In public health, where decisions must be justified transparently and democratically, opacity undermines accountability. Moreover, AI tools are often developed within siloed technical environments that are disconnected from the institutional realities of public health systems. El-Sayed et al. (3) note that the absence of standardized protocols for validation, deployment, and oversight remains a major barrier to meaningful integration. Without harmonized governance structures, AI risks becoming an add-on technology rather than a coherent component of population health strategy.

## 3 Foundational principles vs. algorithmic logic

### 3.1 Population health and prevention

A defining feature of public health is its focus on populations rather than individuals, with prevention as a central objective. In contrast, many contemporary AI applications emphasize individual-level risk prediction, aligning more closely with paradigms of personalized or precision medicine. While individual risk stratification can inform targeted interventions, an excessive focus on personal vulnerability may divert attention and resources from upstream determinants of health, such as housing, education, labor conditions, and environmental exposures (4). AI aligns more closely with public health prevention when applied to collective risk assessment. Examples include environmental surveillance systems, early warning models for vector-borne diseases, and climate, health monitoring platforms. Machine learning–based dengue forecasting systems demonstrate how AI can support anticipatory action at the population level when embedded within preventive frameworks (5). The public health value of AI therefore depends less on technical novelty and more on whether applications illuminate shared risks and inform structural interventions.

### 3.2 Equity and social justice

Equity is the ethical core of public health practice. AI systems trained on historically biased or incomplete data risk reproducing, and amplifying, existing inequities. Empirical evidence shows that algorithms used in health management can disadvantage marginalized populations when socioeconomic context is inadequately represented. The landmark study by Obermeyer et al. (6) revealed that cost-based proxies in population health algorithms led to systematic racial bias in care allocation. More recent analyses from low- and middle-income settings suggest that “algorithmic prejudice” is not a theoretical concern but a material harm. Models trained predominantly on urban or high-resource data often fail to capture rural disease dynamics, informal care pathways, or gendered patterns of access. Nasir et al. (7) describe this phenomenon as a “digital shadow,” wherein populations already underserved by health systems remain underrepresented in digital infrastructures. Realizing AI's potential to advance equity requires deliberate governance choices: inclusive data practices, routine equity audits, and mechanisms for community participation in model design and evaluation. Equity cannot be retrofitted after deployment; it must be treated as a design principle rather than an external constraint.

### 3.3 Systems thinking and complexity

Public health challenges are embedded within complex adaptive systems that span biological, social, economic, and political domains. In principle, AI's capacity to model interactions across multiple levels makes it well suited to such complexity (8). Systems-oriented applications, such as simulations of health system resilience or policy impact modeling, illustrate this potential. In practice, however, computational outputs require contextual interpretation. Over-reliance on algorithmic authority risks oversimplifying social dynamics and marginalizing experiential and local knowledge. Recent frameworks emphasize the importance of “human-in-the-loop” approaches, in which AI supports, but does not replace, professional judgment and community insight. Trust in public health institutions depends not on automation alone, but on transparent deliberation and shared decision-making.

## 4 Strengths and structural limitations of AI in public health

AI offers clear advantages in speed, scalability, and pattern recognition. These strengths are particularly valuable during emergencies, where timely situational awareness can save lives. Real-time infectious disease monitoring platforms, including wastewater surveillance and digital signal detection systems, have demonstrated promising accuracy in outbreak identification (9). Nevertheless, these benefits are constrained by data quality, generalizability, and infrastructure requirements. Algorithms that perform well in one epidemiological or sociopolitical context often fail when transferred elsewhere. The persistent “myth of neutrality” in AI can obscure the value-laden choices embedded in model development, from variable selection to outcome definition (10). Additionally, the high costs of digital infrastructure and the opacity of deep learning models continue to limit adoption, particularly in resource-constrained settings (17). These limitations underscore the need to evaluate AI not only as a technical artifact but as a sociotechnical system shaped by power relations, institutional capacity, and governance norms.

## 5 Discussion: from technological enthusiasm to value-driven governance

The integration of AI into public health represents a critical inflection point for the discipline. Whether AI becomes a foundational advance or a technocratic distraction depends on how it is governed, interpreted, and deployed. Conceptualizations such as “precision public health” offer a pathway forward, provided they prioritize social gaps and population-level benefit rather than narrow clinical optimization (11). Several principles emerge from this analysis. First, transparency and explainability are prerequisites for legitimacy. AI systems influencing public policy must be interpretable to decision-makers and understandable to the communities they affect (15). Second, equity must be embedded throughout the AI lifecycle, from data collection to post-deployment monitoring. Initiatives such as the NIH All of Us framework illustrate how inclusive data strategies can support more just outcomes (12). Finally, robust regulatory and ethical frameworks are essential. The WHO's 2025 guidance on LMMs emphasizes accountability, safety, and human oversight as non-negotiable components of AI for health. Such frameworks should be viewed not as barriers to innovation but as enablers of responsible, legitimate, and trustworthy public health practice.

## 6 Conclusion and recommendations

Artificial intelligence represents a critical juncture for contemporary public health, but its significance lies not in computational sophistication alone; rather, it depends on how it is governed, interpreted, and aligned with the discipline's normative foundations. As this article has argued, AI can constitute a foundational shift only when it reinforces public health commitments to prevention, equity, transparency, and collective accountability. When adopted uncritically, however, AI risks narrowing public health practice into a technocratic enterprise—one that privileges predictive efficiency over social context, algorithmic authority over democratic deliberation, and data availability over moral judgment. Public health decisions shape population trajectories and distributive outcomes; delegating such decisions to opaque systems without robust oversight threatens institutional legitimacy and public trust. Recent scholarship underscores that responsible AI integration must be explicitly equity-oriented, participatory, and embedded within public governance structures, rather than operating as a parallel technical domain (13, 14). Moving forward, public health institutions must shift from technological enthusiasm to value-driven AI governance. Equity should be embedded throughout the entire AI lifecycle, from inclusive data generation and model design to continuous monitoring for bias and unintended harm, particularly in low-resource and marginalized settings. Transparency and explainability must be treated as democratic prerequisites rather than optional technical features, especially when algorithmic outputs inform policy decisions, prioritization, or resource allocation. Investment priorities should favor AI applications that strengthen population-level prevention and illuminate upstream social and environmental determinants of health, rather than narrowly focusing on individual risk stratification. Finally, participatory and human-in-the-loop approaches are essential to ensure that professional judgment, community knowledge, and ethical reasoning remain central to decision-making. As recent public health ethics literature emphasizes, fairness, accountability, and justice are not external constraints on innovation but conditions for its legitimacy and sustainability. When guided by these principles, AI can enhance public health's capacity to address complexity and inequity; without them, it risks becoming a technocratic distraction that obscures the very values public health exists to protect.

## References

[1] Wang Y Song Y Wang Y Yu L Wang J. Ethics and governance of artificial intelligence for health: guidance on large multi-modal models. Chinese Med Ethics. (2024) 100. 10.3760/cma.j.cn112150-20240709-00548 40518431

[2] Topol EJ. High-performance medicine: the convergence of human and artificial intelligence. Nat Med. (2019) 25:44–56. doi: 10.1038/s41591-018-0300-7 30617339

[3] El-Sayed AA Reiss UM Hanna D Bolous NS. The role of public health in rare diseases: hemophilia as an example. Front Public Health. (2025) 13:1450625. doi: 10.3389/fpubh.2025.1450625 40182514 PMC11965367

[4] Frieden TR. A framework for public health action: the health impact pyramid. Am J Public Health. (2010) 100:590–5. doi: 10.2105/AJPH.2009.185652 20167880 PMC2836340

[5] Sophia Y Roxy MK Murtugudde R Karipot A Sapkota A Dasgupta P . Dengue dynamics, predictions, and future increase under changing monsoon climate in India. Sci Rep. (2025) 15:1637. doi: 10.1038/s41598-025-85437-w 39837878 PMC11750985

[6] Obermeyer Z Powers B Vogeli C Mullainathan S. Dissecting racial bias in an algorithm used to manage the health of populations. Science. (2019) 366:447–53. doi: 10.1126/science.aax2342 31649194

[7] Nasir M Siddiqui K Ahmed S. Ethical-legal implications of AI-powered healthcare in critical perspective. Front Artif Intell. (2025) 8:1619463. doi: 10.3389/frai.2025.1619463 40673212 PMC12263600

[8] Greenhalgh T Papoutsi C. Studying complexity in health services research: desperately seeking an overdue paradigm shift. BMC Med. (2018) 16:95. doi: 10.1186/s12916-018-1089-4 29921272 PMC6009054

[9] Alwakeel MM. AI-assisted real-time monitoring of infectious diseases in urban areas. Mathematics. (2025) 13:1911. doi: 10.3390/math13121911

[10] Joseph J. Algorithmic bias in public health AI: a silent threat to equity in low-resource settings. Front Public Health. (2025) 13:1643180. doi: 10.3389/fpubh.2025.1643180 40771228 PMC12325396

[11] Khoury MJ Iademarco MF Riley WT. Precision public health for the era of precision medicine. Am J Prev Med. (2015) 50:398. doi: 10.1016/j.amepre.2015.08.031 26547538 PMC4915347

[12] Budhu JA Anderson N Branson C Dy-Hollins ME Jimenez-Gomez A Nearing K . Health equity considerations in the age of artificial intelligence. Neurology. (2025) 105:e214356. doi: 10.1212/WNL.0000000000214356 41264894 PMC12636768

[13] Dankwa-Mullan I. Health equity and ethical considerations in using artificial intelligence in public health and medicine. Prevent Chronic Dis. (2024) 21:E64. doi: 10.5888/pcd21.240245 39173183 PMC11364282

[14] Gozum IEA Flake CCD. Human dignity and artificial intelligence in healthcare: a basis for a Catholic ethics on AI. J Relig Health (2024) 2024:1–24. doi: 10.1007/s10943-024-02206-1 39730882

[15] Hanna MG Pantanowitz L Dash R Harrison JH Deebajah M Pantanowitz J . Future of artificial intelligence-machine learning trends in pathology and medicine. Mod Pathol. (2025) 38:100705. doi: 10.1016/j.modpat.2025.100705 39761872

[16] Mittelstadt BD Allo P Taddeo M Wachter S Floridi L. The ethics of algorithms: mapping the debate. Big Data & Soc. (2016) 3:2053951716679679. doi: 10.1177/2053951716679679

[17] Reddy S Allan S Coghlan S Cooper P. A governance model for the application of AI in health care. J Am Med Inform Assoc. (2020) 27:4917. doi: 10.1093/jamia/ocz192 31682262 PMC7647243

